# Supplementary material for: Circulating microRNA miR-425-5p Associated with Brain White Matter Lesions and Inflammatory Processes
Source: Int J Mol Sci. 2024 Jan 10;25(2):887. doi: 10.3390/ijms25020887 (PMC10815886; doi:10.3390/ijms25020887)
Supplement: Supplementary file 1 [file ijms-25-00887-s001.zip › supplement_WMH_miRNA.pdf]

Supplementary Methods and Figures

Circulating microRNA miR-425-5p as potential regulator of brain white matter lesions through inflammatory processes

Supplementary Methods

Verbal memory score NAI (Nuremberg Age Inventory)

Participants are asked to memorize words read to them (immediate verbal memory score) and are later on presented a list containing the same words and additional distractor words. The number of correctly identified words is summarized to a sum score minus the number of wrongly identified distractor words (delayed verbal memory score). The resulting scores can be interpreted as a mesure of short- and long-term memory capacity.

Brain Imaging Data

For structural MRI data, participants were scanned with a 1.5 Tesla MRI (MAGNETOM Avanto; Siemens Healthcare, Erlangen, Germany) with a T1-weighted magnetization-prepared rapid acquisition gradient echo (MPRAGE) sequence and the following parameters: axial plane, repetition time = 1900 ms, echo time = 3.4 ms, flip angle = 15°, original resolution = 1.0 × 1.0 × 1.0 mm3, matrix = 256 × 176, bandwidth = 130 Hz/Pixel [86].

WML were segmented by the lesion growth algorithm as implemented in the LST toolbox version 3.0.0 (www.statistical-modelling.de/lst.html) for SPM (Statistical Parametric Mapping, https://www.fil.ion.ucl.ac.uk/spm/) using both the T1-weighted and the FLAIR MRI sequences [88]. We set the initial threshold kappa to 0.25 and used a threshold of 0.5 to generate binary lesion maps based on the obtained probability maps to be able to extract the total lesion volume and the number of lesions present.

Plasma-circulating miRNA data

Blood samples in TREND-0 were taken from the cubital vein. Serum and plasma samples were stored at -80°C in the Integrated Research Biobank of the University Medicine Greifswald and used in accordance with its regulations.

MiRNA levels were measured in a subsample of TREND-0 participants (n = 708). These individuals have been selected from a TREND-0 subsample of 1000 individuals with additional available genetic and gene-expression information to obtain a maximum overlap of different OMICS layers. As this sample was used for gene-expression analyses, subjects with known

diabetes have been excluded. The subjects for miRNA analysis have been selected to reflect the same age, sex and BMI distribution as in the larger subsample of 1000 individuals. MiRNAs were prepared from 200µl EDTA plasma using the miRCURY™ RNA Isolation Kit –Biofluids (Qiagen, Hilden, Germany) and were measured in two batches (batch 1: n = 371; batch 2: n = 337).

The laboratory workflow involved several quality control steps. First, to ensure high and reproducible RNA yield from EDTA-plasma samples, bacteriophage MS2 carrier RNA and the Spike-Ins UniSp2, UniSp4, and UniSp5 were added to each sample during the purification procedure. Reverse transcription reactions were performed and monitored with the Spike-ins UniSp6 and Cel-miR-39 using the Universal cDNA Synthesis Kit II (Qiagen, Hilden, Germany) according to the manufacturer's instructions. Before using RNA samples for miRNA profiling, the presence of Spike-Ins (UniSp2, UniSp4, UniSp5), yield of typical plasma miRNAs, absence of PCR inhibitors (UniSp6, Cel-miR-39) as well as hemolysis in the samples was assessed by use of a microRNA QC PCR Panel V1.M (Qiagen, Hilden, Germany). Samples which did not pass the quality control were excluded from further processing.

For RT-qPCR based miRNA analysis the Serum/ Plasma Focus microRNA PCR Panel (Qiagen, Hilden, Germany) V3.M and V4.M were used, covering 179 miRNAs. The qPCR of the first batch of TREND-0 miRNA samples was performed on the 7900HT Real-Time PCR system and the second batch was performed on the QuantStudio™ 12K Flex Real-Time PCR system (both Thermo Fisher scientific, Waltham, USA). Cycling parameters as recommended by Qiagen were employed in 42 amplification cycles. The amplification curves were analyzed using the SDS 2.4 and Quantstudio Real-time PCR System software version 1.3 (both Thermo Fisher Scientific, Waltham, USA) for determination of Cycle-treshold (Ct) and for melting curve analysis. For each batch, a miRNA was selected for further analysis if Ct ≤ 37 was detected in at least 40% of the samples. In order to consider the influence of technical parameters, the Ct values of synthetic spiked-in miRNAs — monitoring the efficiency of miRNA extraction (UniSp2 and the difference between Ct values of UniSp4 and UniSp2) — the interplate calibrator (UniSp3), as well as the storage time of the biosamples (dt_biobank) were regressed out of the data. Linear regression was performed for each miRNA according to the model ΔCt ~ UniSp2 + UniSp4-UniSp2 + UniSp3 + dt_biobank, which treats the ΔCt values as dependent variables and the technical parameters as independent variables. The resulting residuals were used as independent variables in later models to detect associations between miRNAs and phenotypes of interest. A batch was included in the analysis for a specific miRNA if at least 100 subjects contained a valid measurement of the respective miRNA (see supplementary Table S1 for a complete list of miRNAs used).

Genome-wide SNP chip and APOE genotype

Genome-wide SNP information was taken from the genetic data in SHIP. Genotyping of a subset of the SHIP-TREND subjects (n = 986 subjects that fasted for at least 10 hours prior to blood sampling and had serum fasting glucose levels ≤ 8mmol/l; also including the miRNA

sample) was performed using the Illumina Infinium HumanOmni 2.5 Bead Chip. The remaining SHIP-TREND sample (n = 3134) was genotyped using the Illumina Infinium GSA. Imputation of genotypes was performed using the HRCv1.1 reference panel and the Eagle and minimac3 software implemented in the Michigan Imputation Server for pre-phasing and imputation, respectively. For more details, see Völzke and colleagues [70]Klicken oder tippen Sie hier, um Text einzugeben.. SNPs with a Hardy–Weinberg-Equilibrium *p*-value < 0.0001, a call rate < 0.95, and a MAF < 1% were removed before imputation.

Genome-wide SNP information was taken from the genetic data in TREND-0 (see supplementary methods). The APOE genotypes were determined based on the two SNPs rs429358 (C; T) and rs7412 (T; C) from the resulting imputation (imputation quality > 0.8; Hardy–Weinberg Equilibrium, p > 0.05) [87]. As we used the data from the genome-wide SNP chip instead of strand-specific genotyped SNPs for the determination of APOE status, two ambiguous SNP combinations occurred where APOE ε2/ε4 and ε1/ε3 could not be distinguished from each other (http://www.snpedia.com/index.php/APOE; accessed on June 27th 2022). Those participants in TREND-0 were excluded from the APOE analyses (n = 99). Subjects were defined as APOE ε4 carriers if they had at least one ε4 allele.

Additional Variables

Current depressive symptoms were assessed in TREND-0 using the patient health questionnaire (PHQ-9), a 9-item self-report questionnaire with high reliability and validity with higher values reflecting a higher burden of depressive symptoms. Education measured as the number of schooling years was divided into three categories according to the German school system: less than 10 years, exactly 10 years, and more than 10 years. Smoking status was coded as never/former/current smoker and body mass index (BMI) was calculated as (weight in kg)/(height in m)2. HbA1c was measured by high-performance liquid chromatography with spectrophotometric detection (Diamat Analyzer; Bio-Rad, Munich, Germany). Hypertension was defined in case of the intake of antihypertensive medication or systolic/diastolic blood pressure above 140/90 mmHg during the assessment. Haematocrit (HCT) and platelets (PLT) were measured as part of the clinical blood count routine.

Supplementary Figures

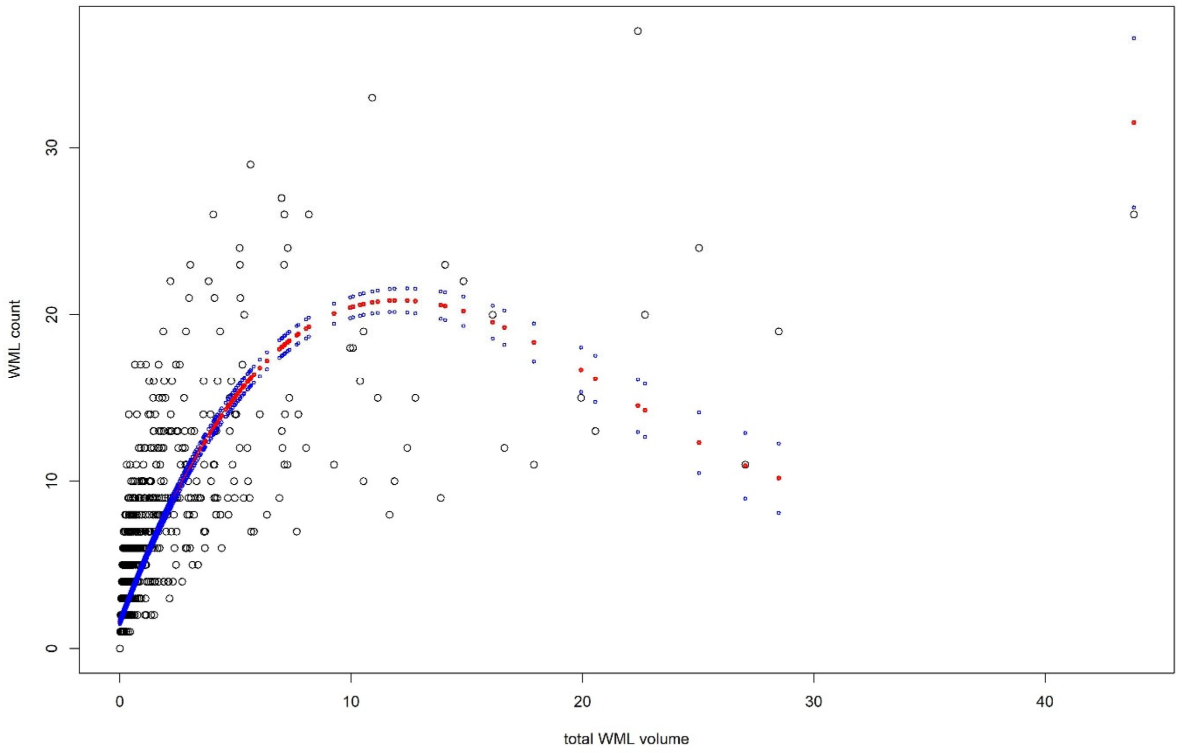

A

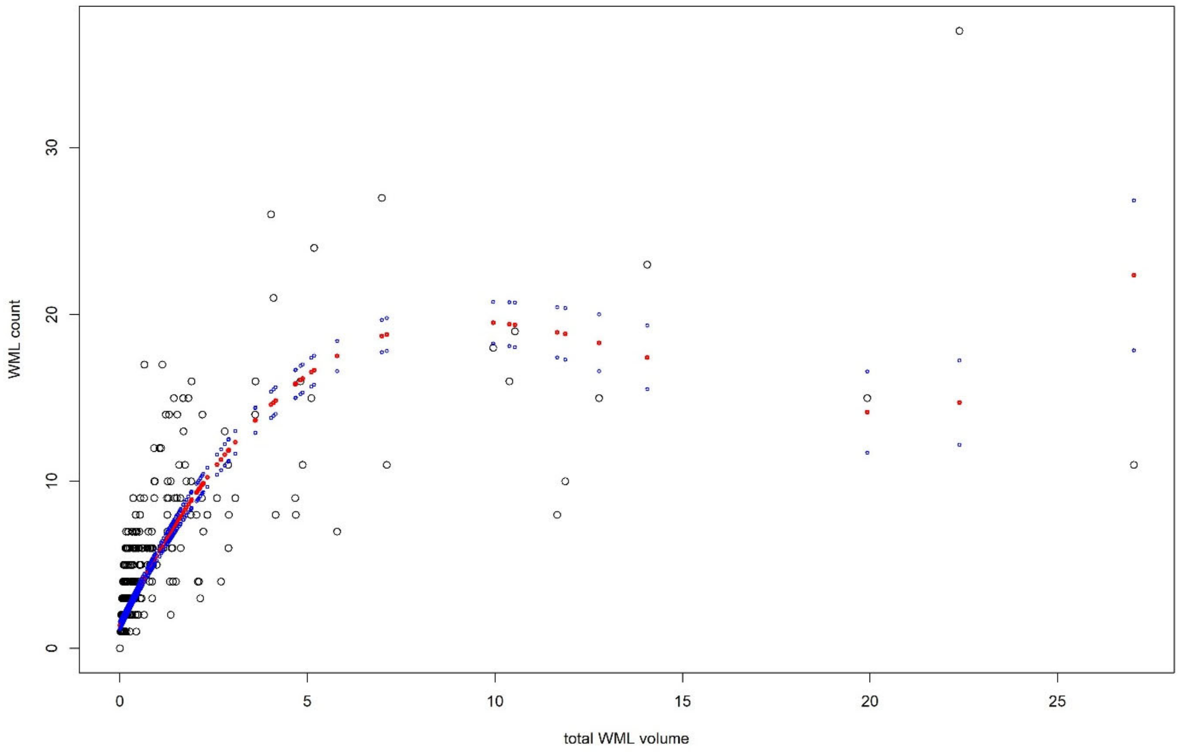

B

Figure S1. A. Non-linear association between total white matter lesion volume and number (count) of white matter lesions in the TREND-0 MRI sample (n = 1854). The red dots represent the predicted non-linear curve, the blue dots the upper and lower bound (95% confidence interval) of the curve. The black dots represent the original data points. Wald test for spline regression coefficients $p <$ 0.001. **B.** Non-linear association between total white matter lesion volume and number (count) of

white matter lesions in the TREND-0 miRNA/MRI sample (n = 647). The red dots represent the predicted non-linear curve, the blue dots the upper and lower bound (95% confidence interval) of the curve. The black dots represent the original data points. Wald test for spline regression coefficients $p < 0.001$.

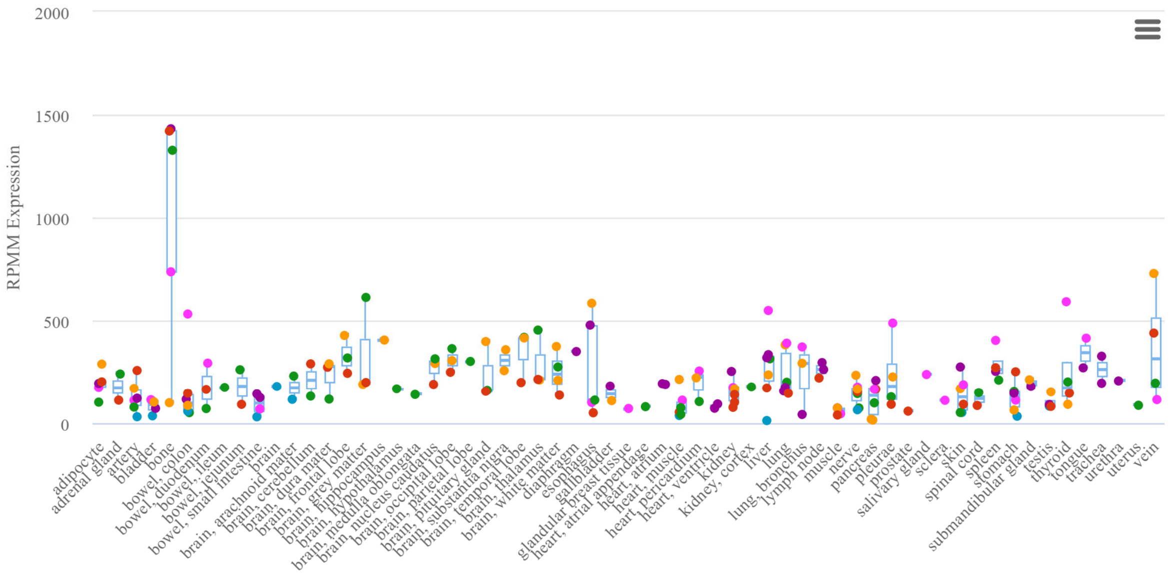

Figure S2. Expression of *hsa*-miR-425-5p across different human tissues (https://ccb-web.cs.uni-saarland.de/tissueatlas2/).

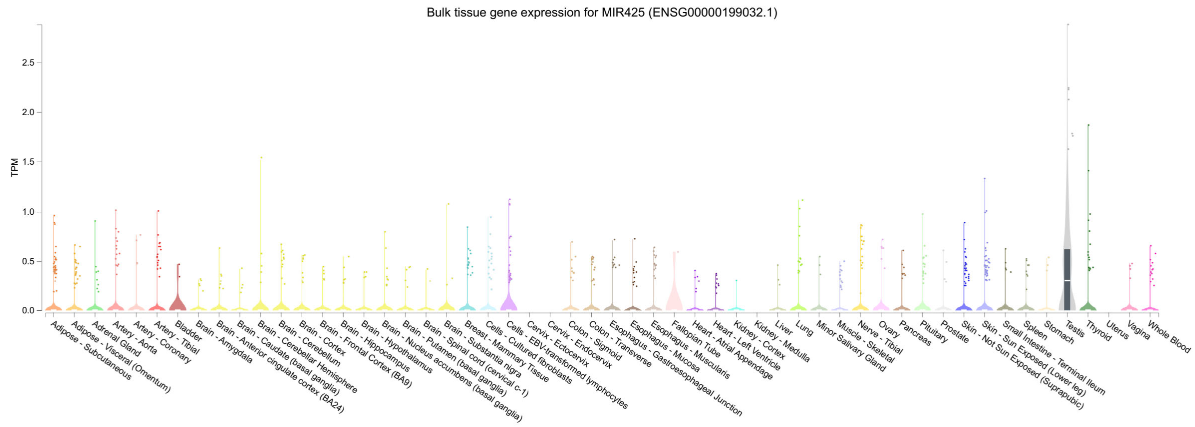

Figure S3. Expression of *MIR425* gene across different human tissues (https://gtexportal.org/home/).

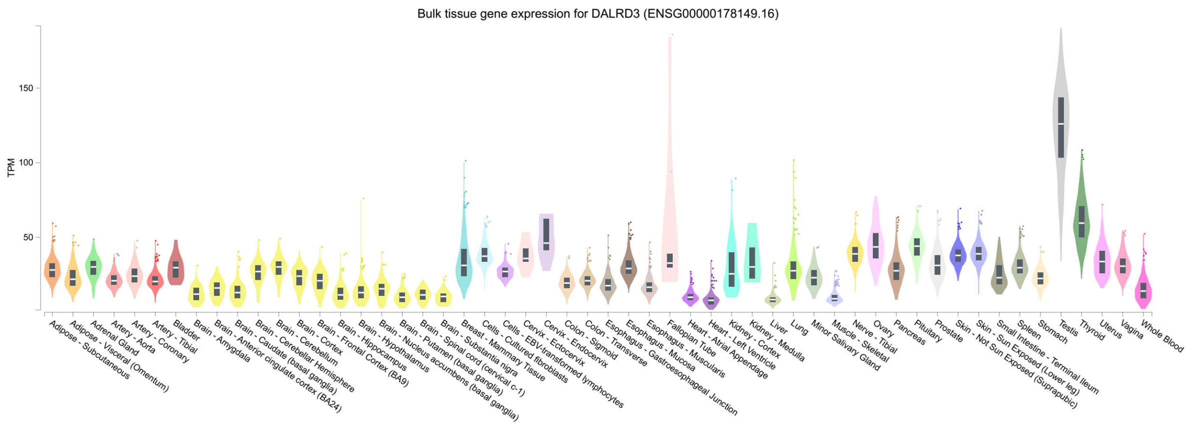

Figure S4. Expression of *DALRD3* gene across different human tissues (https://gtexportal.org/home/).

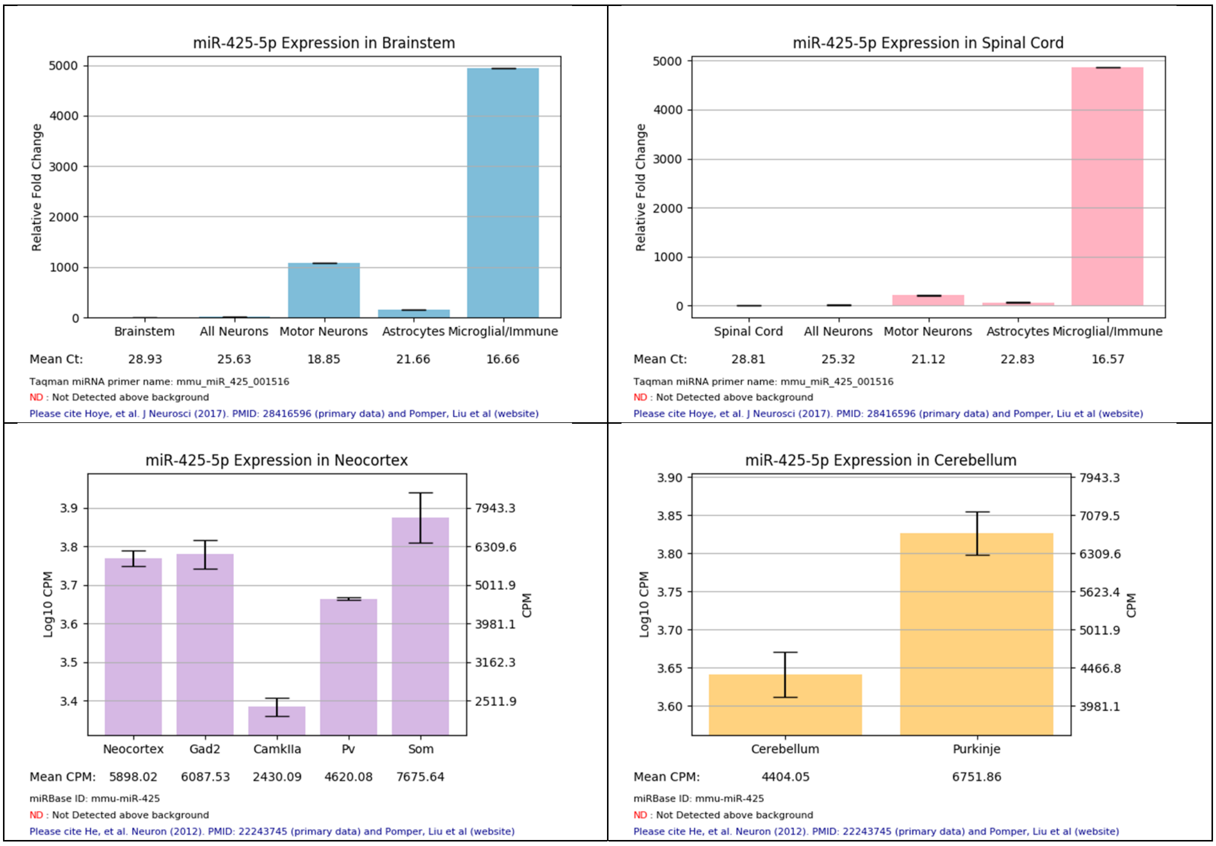

Figure S5. Expression of miR-425-5p in mouse brain from CNS microRNA Profiles (www.mirna.wustl.edu).

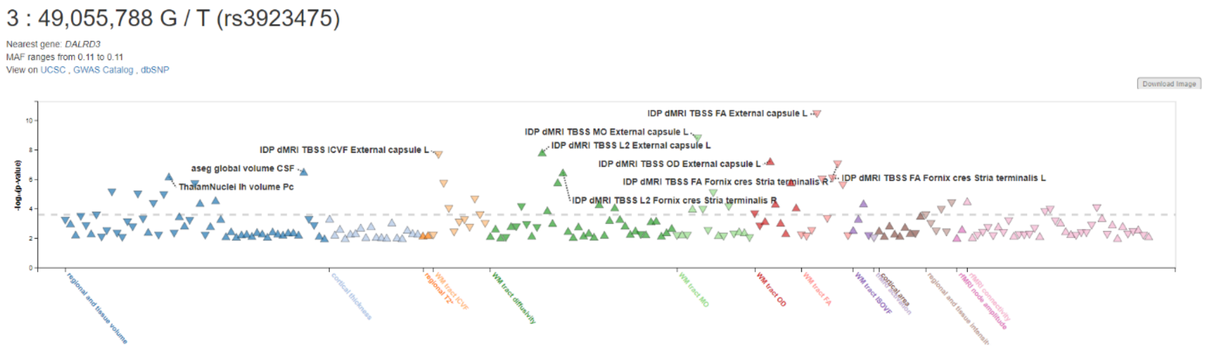

Figure S6. Results for rs3923475 (DALRD3 gene) from Oxford Brain Imaging Genetics server (https://open.win.ox.ac.uk/ukbiobank/big40/).

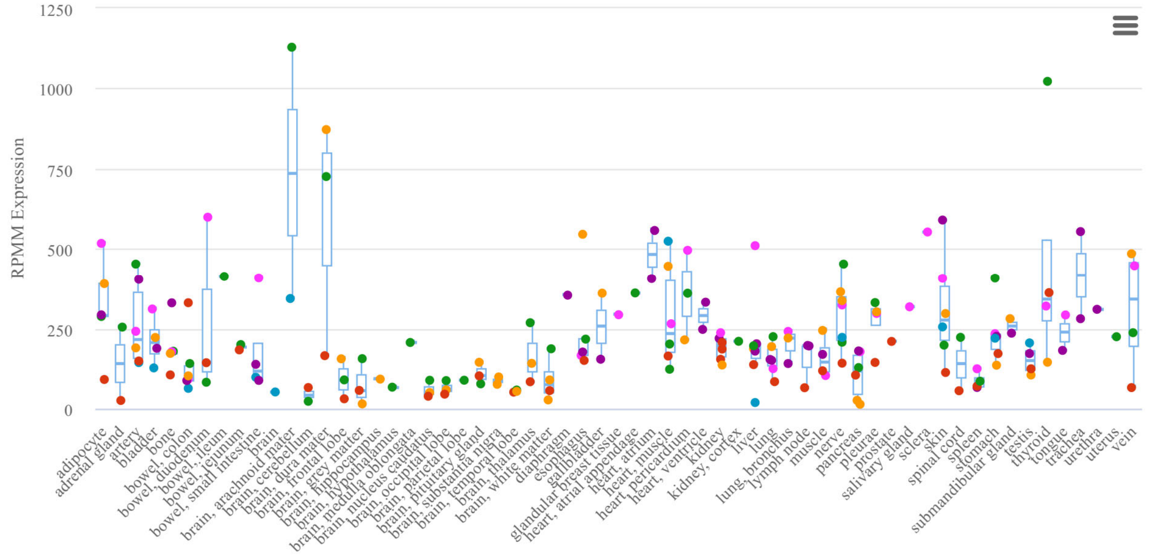

Figure S7. Expression of *hsa*-miR-152-3p across different human tissues (https://ccb-web.cs.uni-saarland.de/tissueatlas2/).

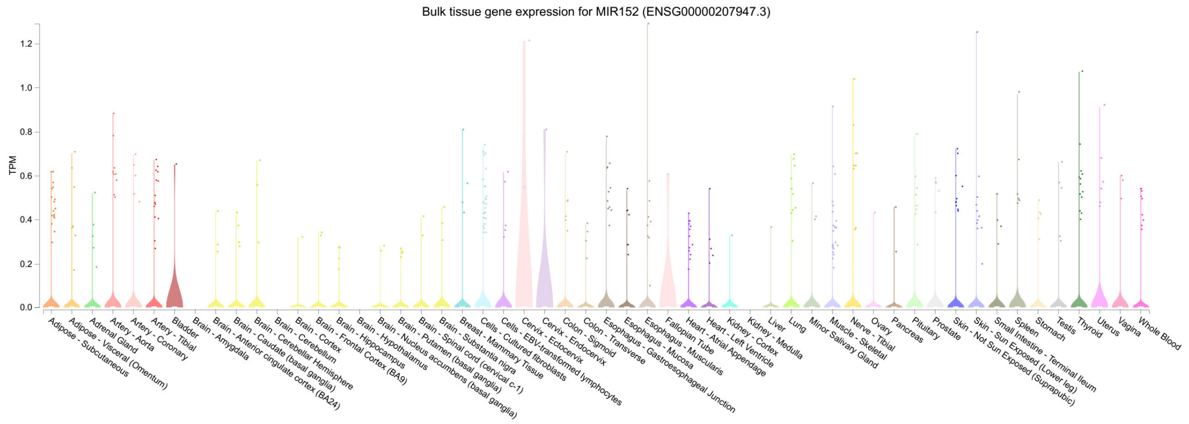

Figure S8. Expression of *MIR152* gene across different human tissues (https://gtexportal.org/home/).

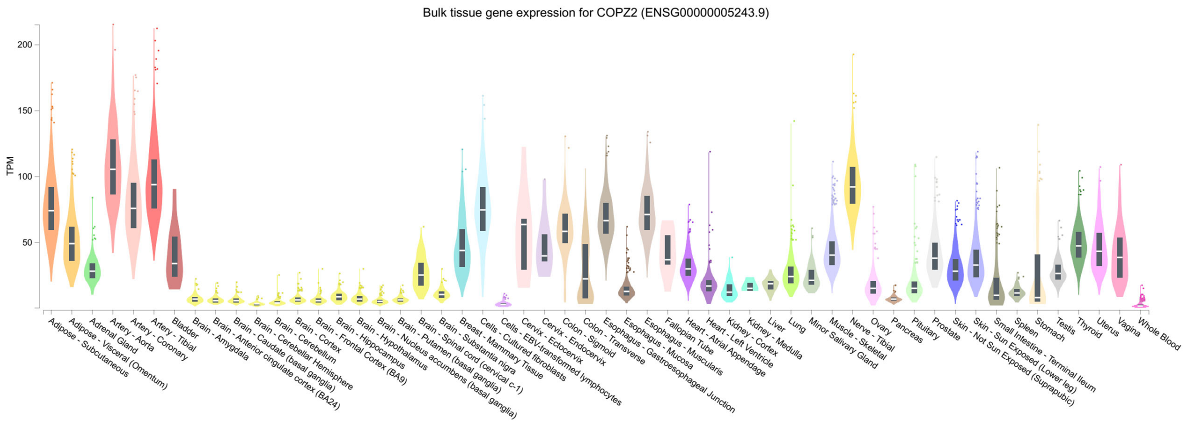

Figure S9. Expression of *COPZ2* gene across different human tissues (https://gtexportal.org/home/).

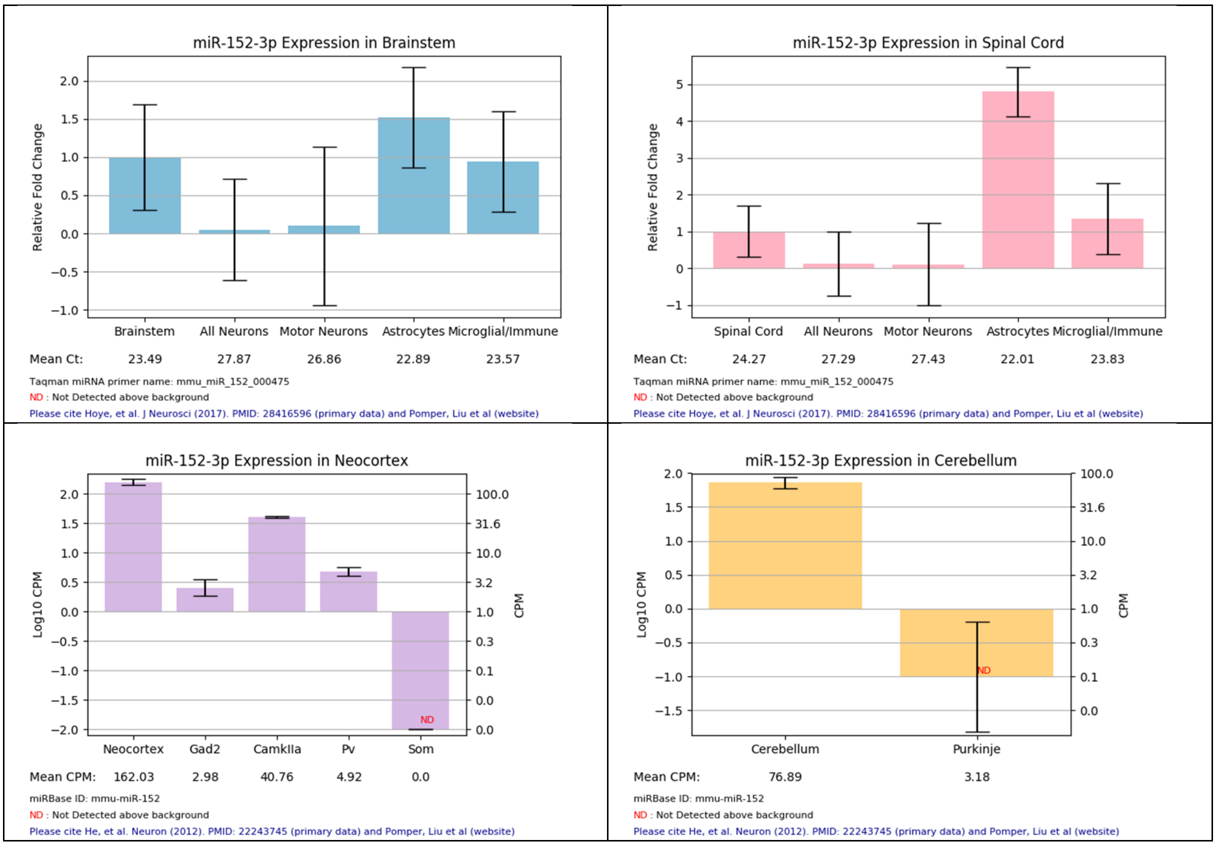

Figure S10. Expression of miR-152-3p in mouse brain from CNS microRNA Profiles (www.mirna.wustl.edu).

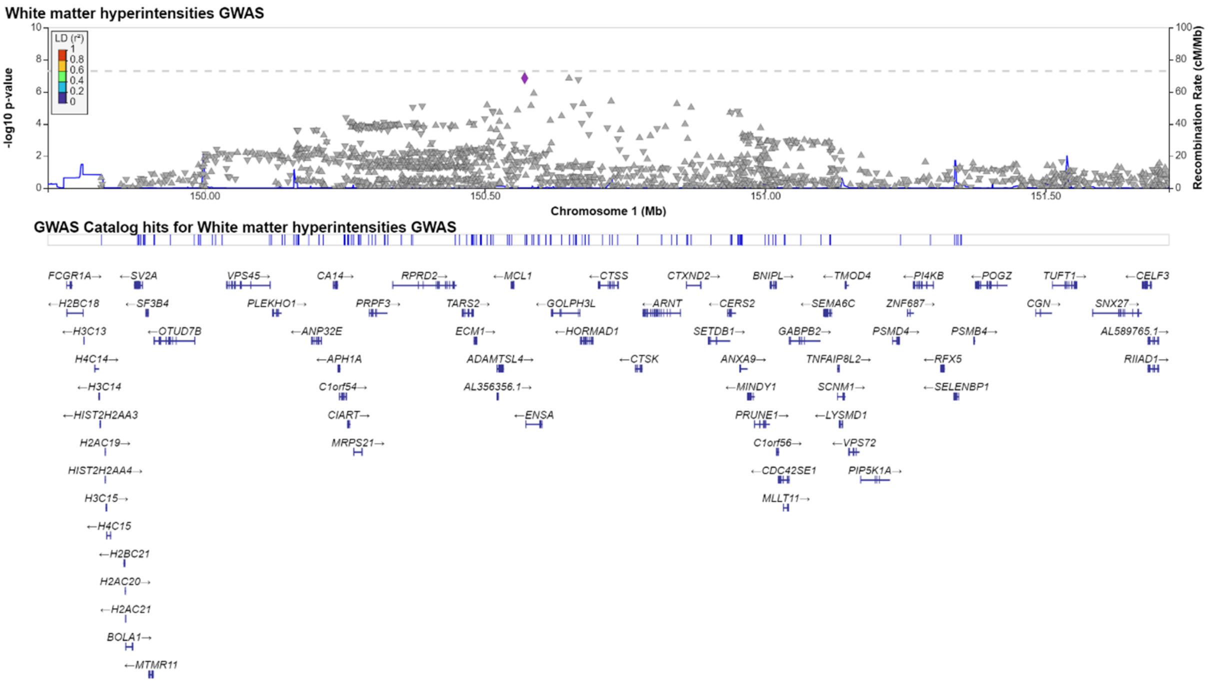
White matter hyperintensities GWAS
-log10 p-value
Recombination Rate (cM/Mb)
LD (r²)
Chromosome 1 (Mb)
150.00
150.50
151.00
151.50
GWAS Catalog hits for White matter hyperintensities GWAS
FCGR1A SV2A VPS45 CA14 RPRD2 MCL1 CTSS CTXND2 BNIPL TMOD4 PI4KB POGZ TUFT1 CELF3
H2BC18 SF3B4 PLEKHO1 PRPF3 TARS2 GOLPH3L ARNT CERS2 SEMA6C ZNF687 CGN SNX27
H3C13 OTUD7B ANP32E ECM1 HORMAD1 SETDB1 GABPB2 PSMD4 PSMB4 AL589765.1
H4C14 APH1A ADAMTSL4 CTSK ANXA9 TNFAIP8L2 RFX5 RIIAD1
H3C14 C1orf54 AL356356.1 MINDY1 SCNM1 SELENBP1
HIST2H2AA3 CIART ENSA PRUNE1 LYSMD1
H2AC19 MRPS21 C1orf56 VPS72
HIST2H2AA4 CDC42SE1 PIP5K1A
H3C15 MLLT11
H4C15
H2BC21
H2AC20
H2AC21
BOLA1
MTMR11

A

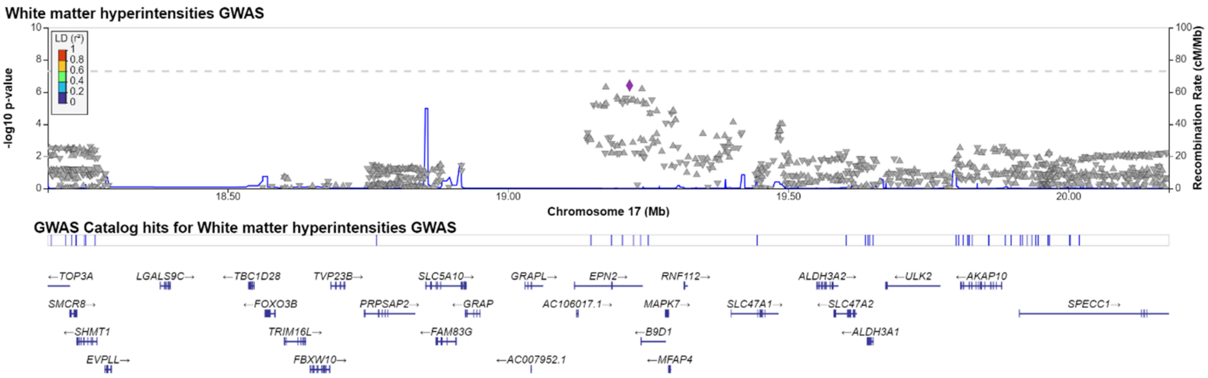
White matter hyperintensities GWAS
-log10 p-value
Recombination Rate (cM/Mb)
LD (r²)
Chromosome 17 (Mb)
18.50
19.00
19.50
20.00
GWAS Catalog hits for White matter hyperintensities GWAS
TOP3A LGALS9C TBC1D28 TVP23B SLC5A10 GRAPL EPN2 RNF112 ALDH3A2 ULK2 AKAP10
SMCR8 FOXO3B PRPSAP2 GRAP AC106017.1 MAPK7 SLC47A1 SLC47A2 SPECC1
SHMT1 TRIM16L FAM83G B9D1 ALDH3A1
EVPLL FBXW10 AC007952.1 MFAP4

B

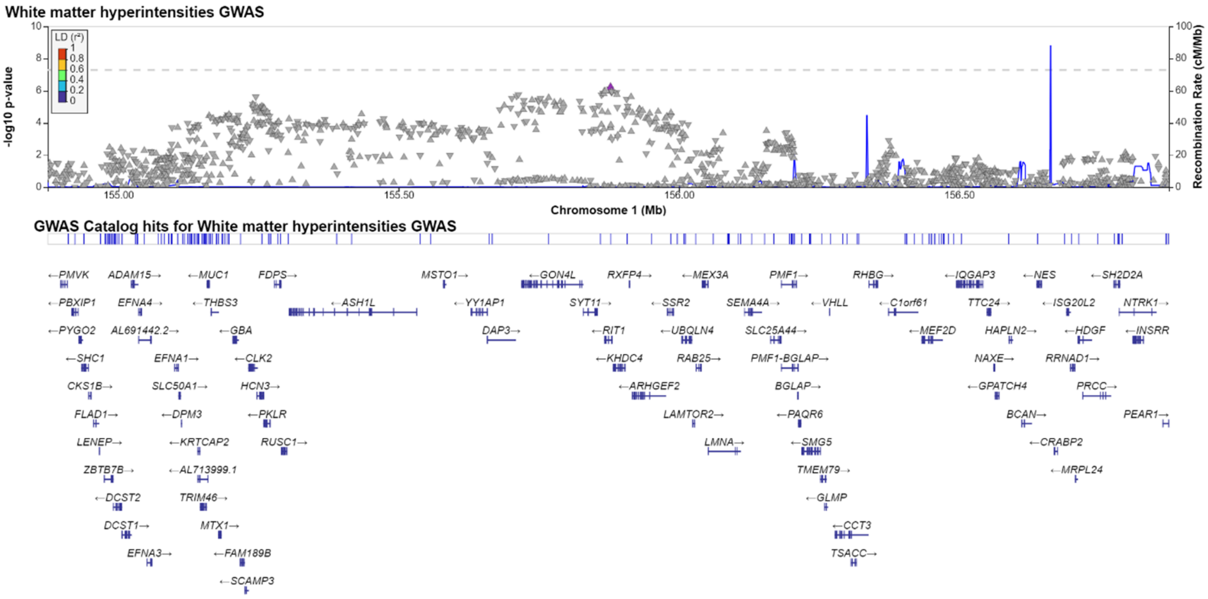
White matter hyperintensities GWAS
-log10 p-value
Recombination Rate (cM/Mb)
LD (r²)
Chromosome 1 (Mb)
155.00
155.50
156.00
156.50
GWAS Catalog hits for White matter hyperintensities GWAS
PMVK ADAM15 MUC1 FDPS MSTO1 GON4L RXFP4 MEX3A PMF1 RHBG IQGAP3 NES SH2D2A
PBXIP1 EFNA4 THBS3 ASH1L YY1AP1 SYT11 SSR2 SEMA4A VHLL C1orf61 TTC24 ISG20L2 NTRK1
PYGO2 AL691442.2 GBA DAP3 RIT1 UBQLN4 SLC25A44 MEF2D HAPLN2 HDGF INSRR
SHC1 EFNA1 CLK2 KHDC4 RAB25 PMF1-BGLAP NAXE RRNAD1
CKS1B SLC50A1 HCN3 ARHGEF2 BGLAP GPATCH4 PRCC
FLAD1 DPM3 PKLR LAMTOR2 PAQR6 BCAN PEAR1
LENEP KRTCAP2 RUSC1 LMNA SMG5 CRABP2
ZBTB7B AL713999.1 TMEM79 MRPL24
DCST2 TRIM46 GLMP
DCST1 MTX1 CCT3
EFNA3 FAM189B TSACC
SCAMP3

C

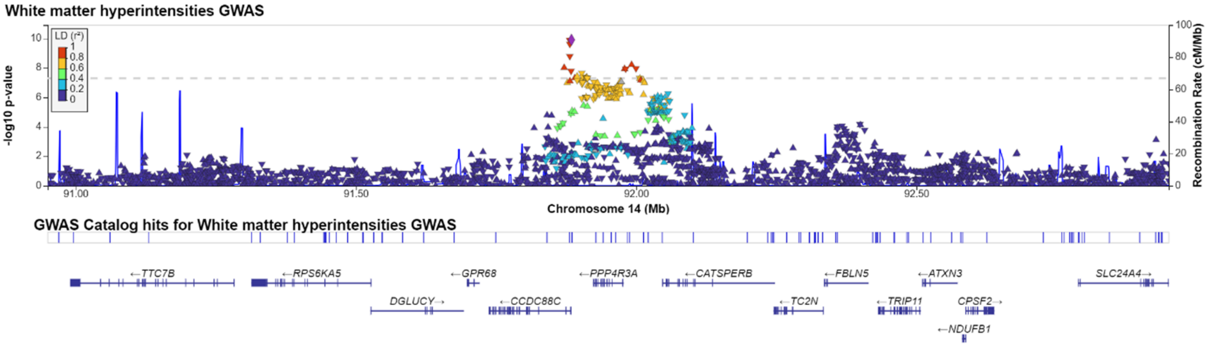

D

Figure S11. A. LocusZoom plot for *CTSS* gene region from white matter hyperintensities GWAS. **B.** LocusZoom plot for *EPN2* gene region from white matter hyperintensities GWAS. **C.** LocusZoom plot for *RIT1* gene region from white matter hyperintensities GWAS. **D.** LocusZoom plot for *PPP4R3A* gene region from white matter hyperintensities GWAS.

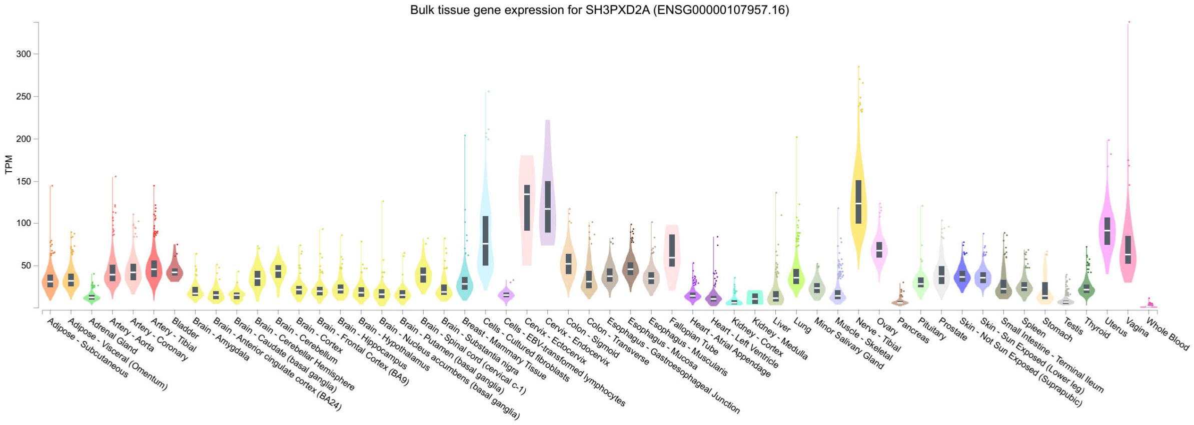

Figure S12. Expression of *SH3PXD2A* gene across different human tissues (https://gtexportal.org/home/).

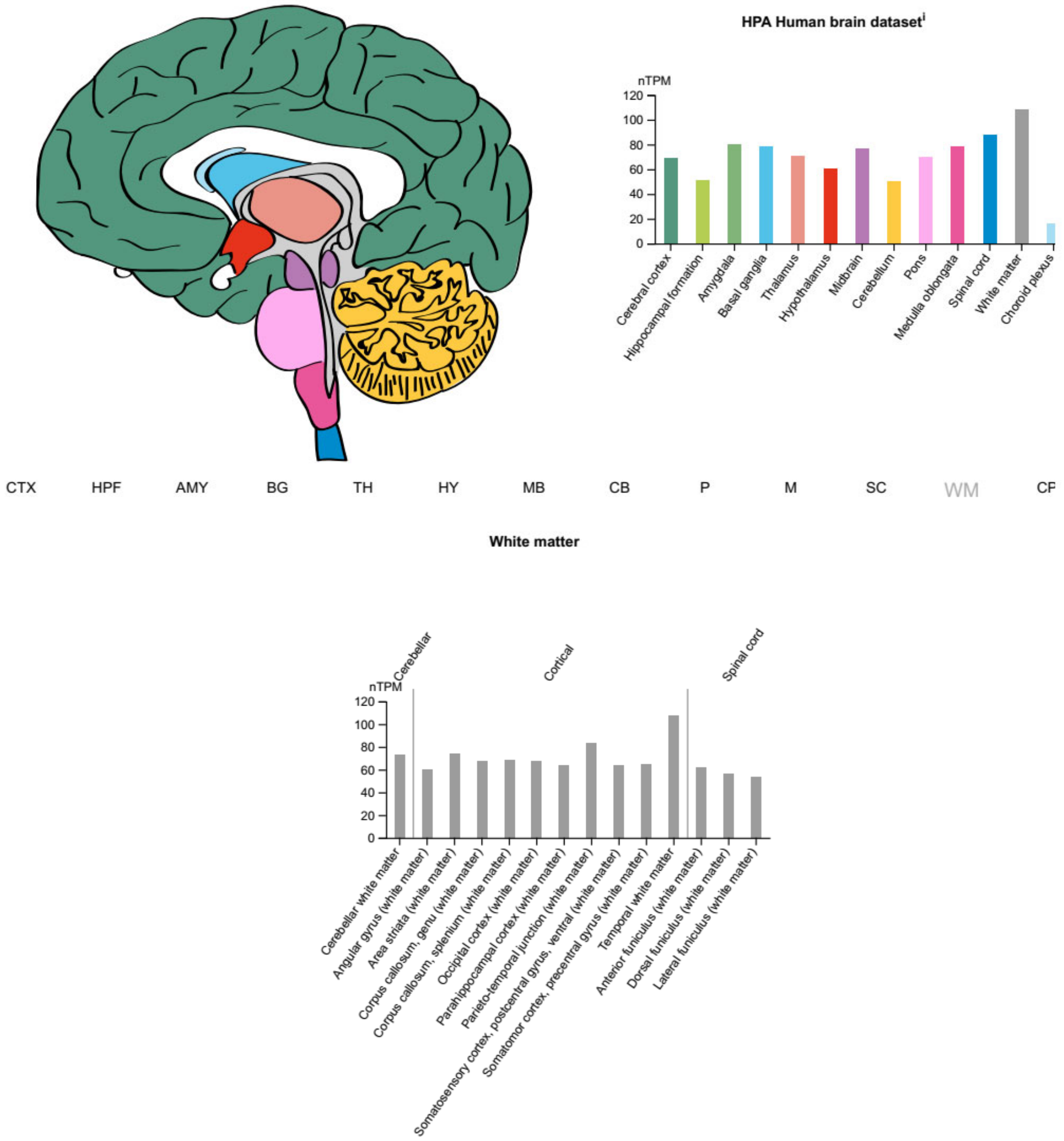

Figure S13. Expression of *SH3PXD2A* gene across different brain regions and tissues (detailed for white matter) from The Human Protein Atlas (https://www.proteinatlas.org/), (nTPM = number of transcripts per million).

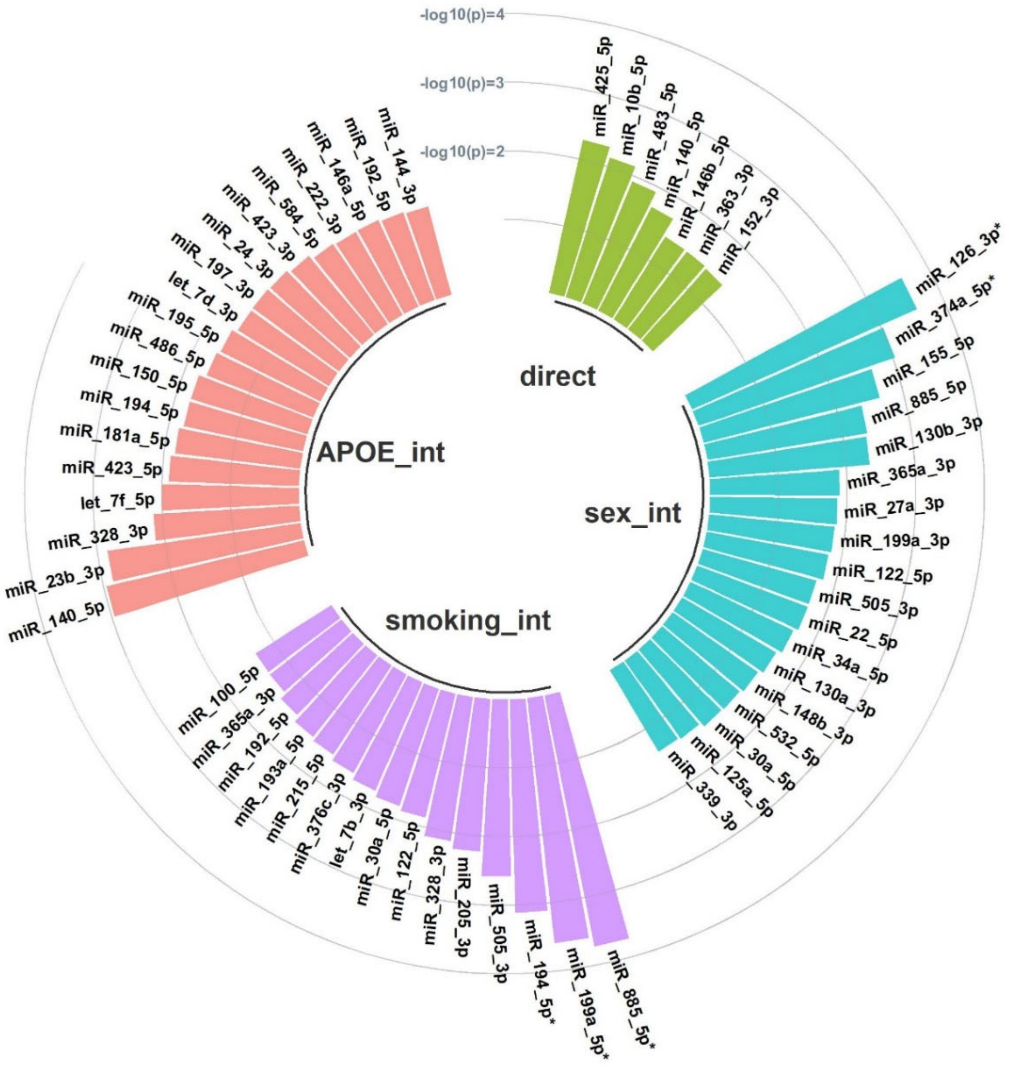

Figure S14. All miRNA with at least nominal significance in the different direct and interaction models on the number of white matter lesions in TREND-0. Direct: direct association between WMLs and miRNAs; sex_int: sex interaction model; smoking_int: smoking interaction model; APOE_int: APOE interaction model.

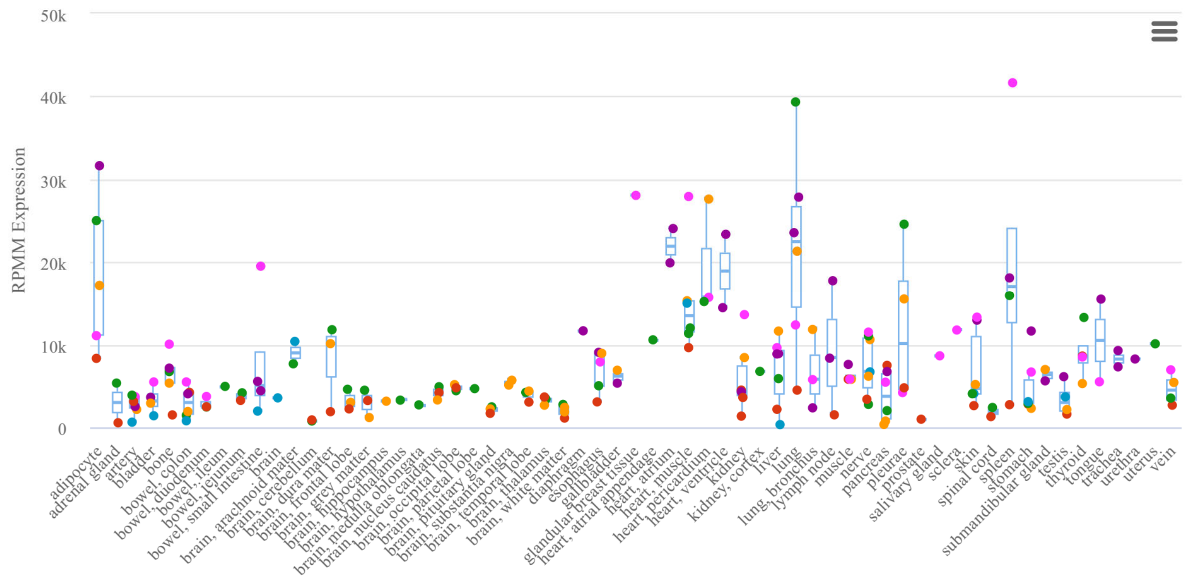

A

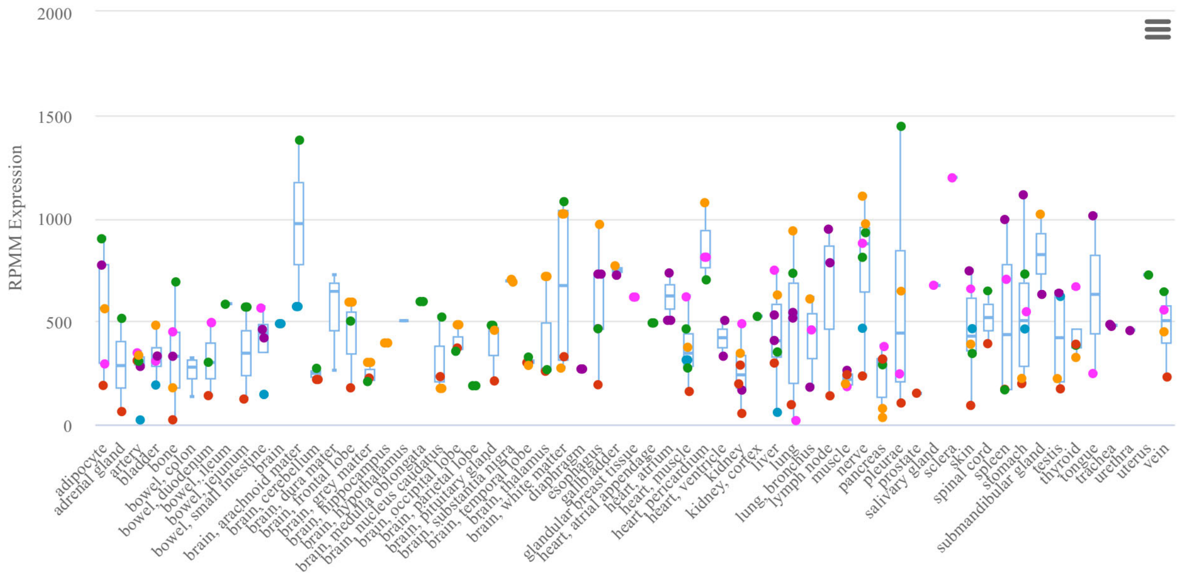

B

Figure S15. A. Expression of miRNA *hsa*-miR-126-3p significant in the sex interaction model in different tissues (https://ccb-web.cs.uni-saarland.de/tissueatlas2/). **B.** Expression of miRNA *hsa*-miR-374a-5p significant in the sex interaction model in different tissues (https://ccb-web.cs.uni-saarland.de/tissueatlas2/).

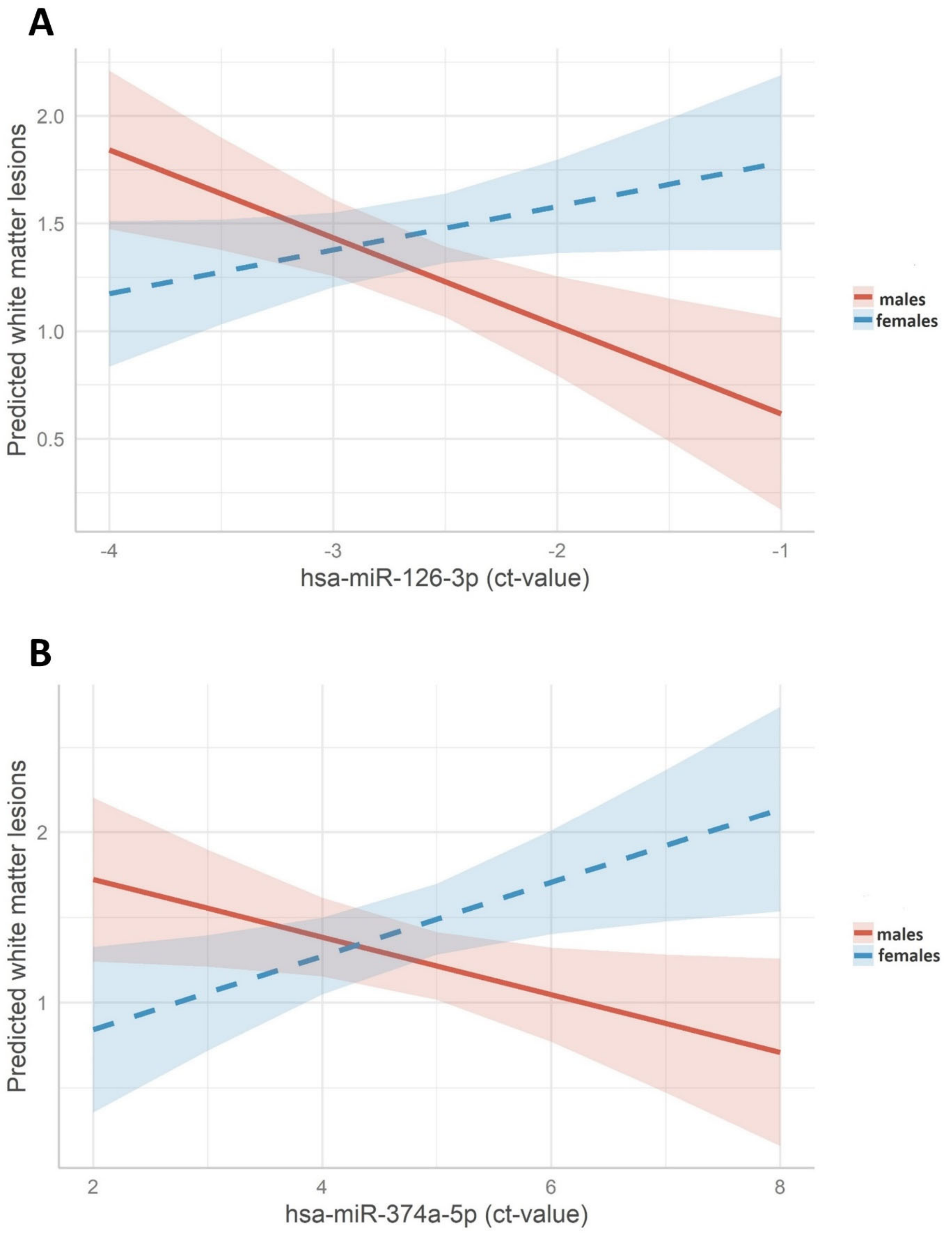

Figure S16. Interaction analysis between sex and miRNA abundance on number of WMLs. (A) *hsa*-miR-126-3p, (B) *hsa*-miR-374a-5p; in both cases lower miRNA abundance was associated with a beneficial outcome for number of white matter lesions in males but with a higher WML burden in females. Values on the x-axis refer to ΔCt-values (ΔCt-values > 0: lower abundance with phenotype, ΔCt-values < 0: higher abundance with phenotype).

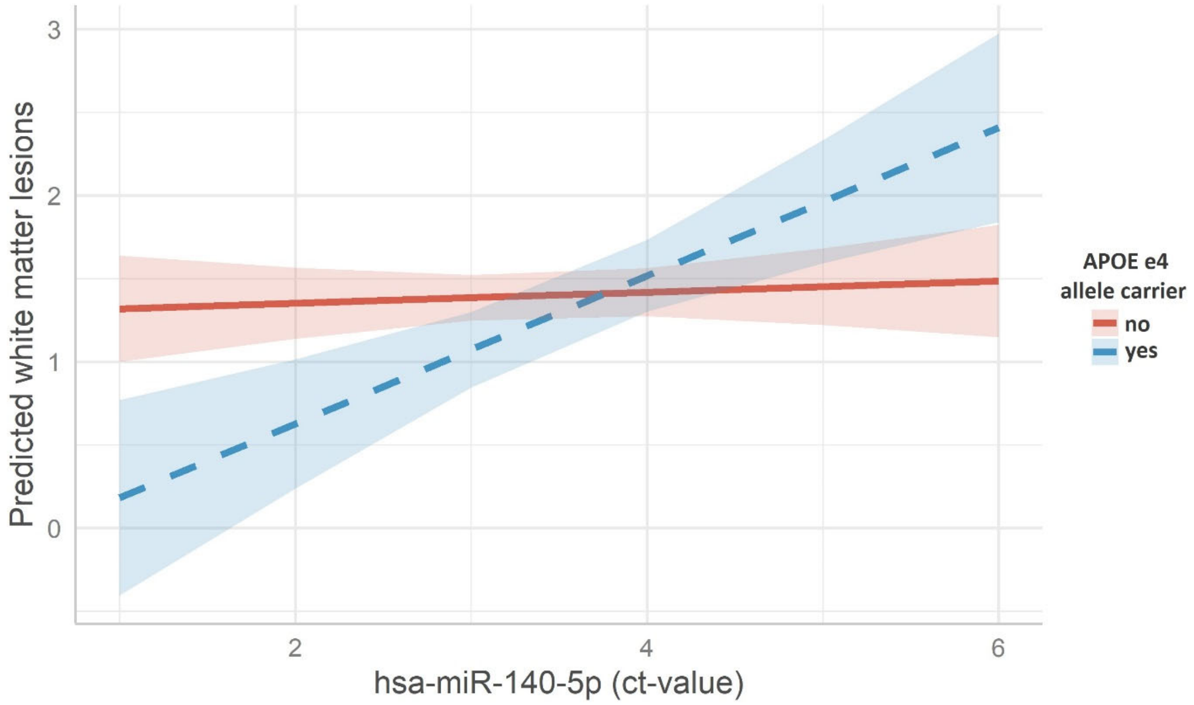

Figure S17. Interaction model between *hsa*-miR-140-5p and *APOE* ε4 carrier status on number of white matter lesions. The number of WMLs has been log-transformed (log(#WMLs + 1)). Values on the x-axis refer to ΔCt-values (ΔCt-values > 0: lower abundance with phenotype, ΔCt-values < 0: higher abundance with phenotype).

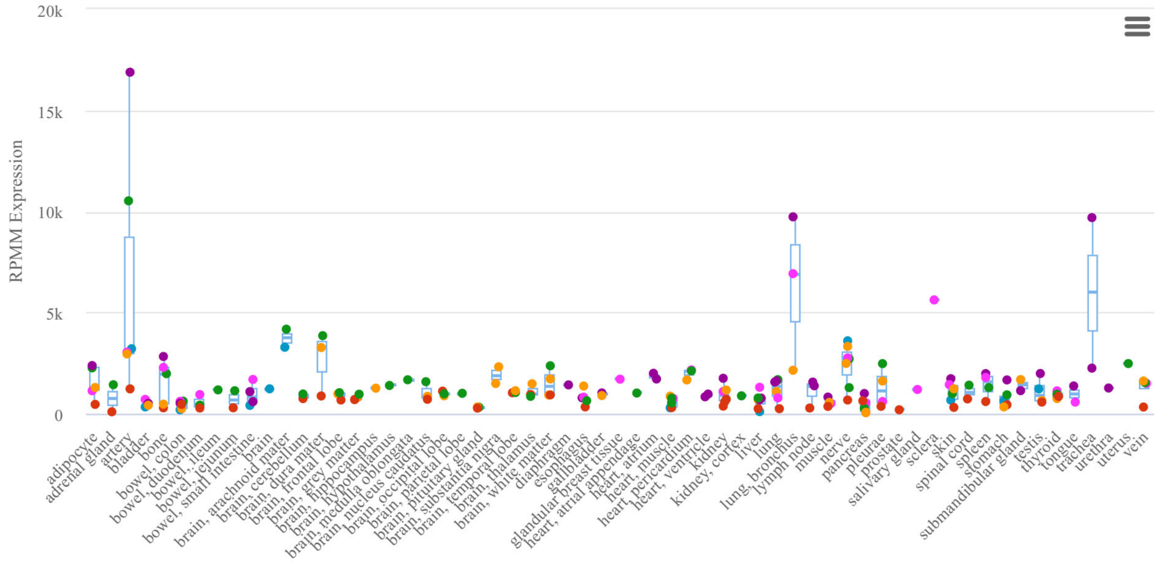

Figure S18. Expression of miRNA *hsa*-miR-140-5p in different tissues (https://ccb-web.cs.uni-saarland.de/tissueatlas2/).

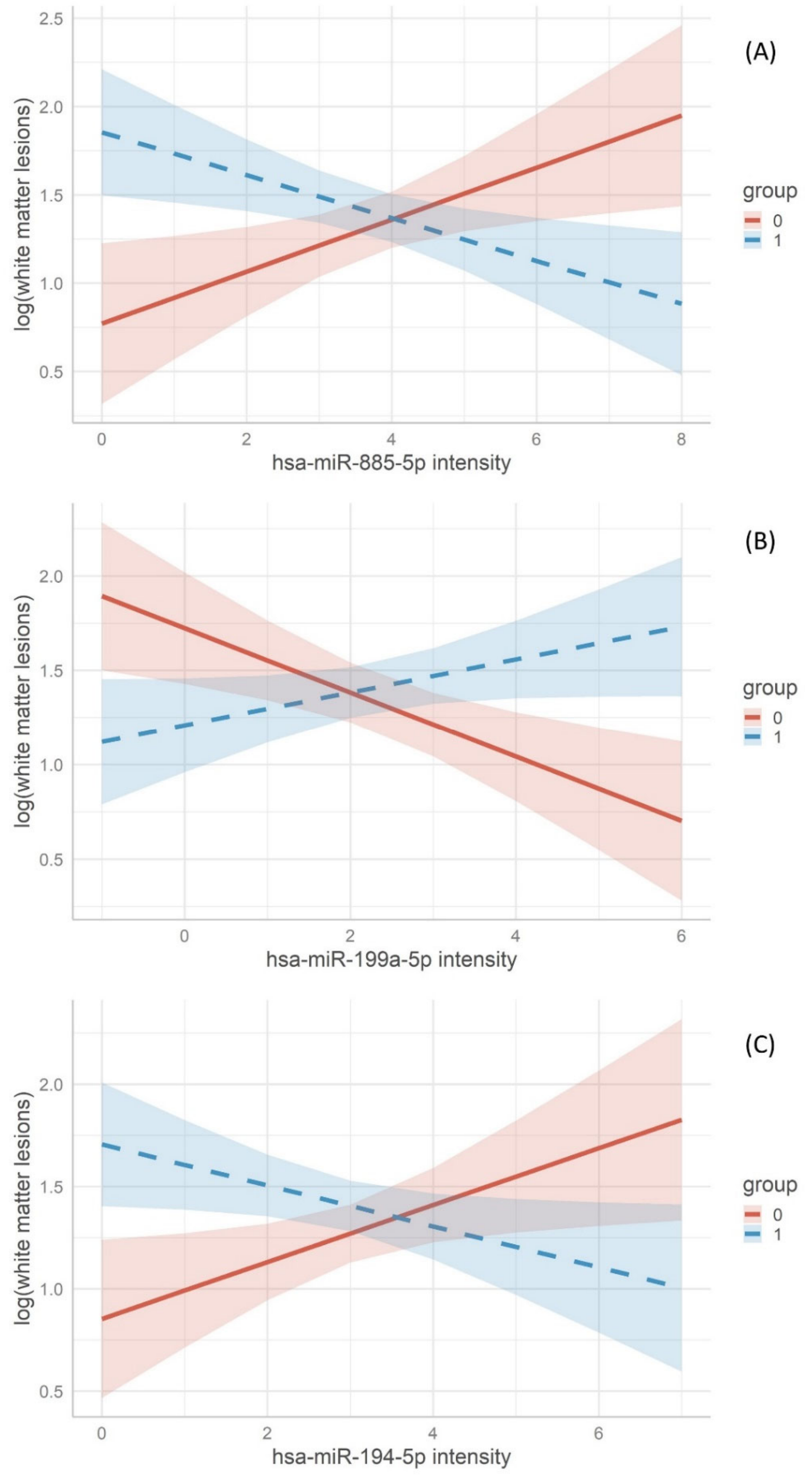

Figure S19. Interaction model between different miRNAs and smoking status on number of white matter lesions. On the x-axis ΔCt-values are displayed. The number of WMLs has been log-transformed (log(#WMLs + 1)). 0: never smokers, 1: ever smokers. Values on the x-axis refer to ΔCt-values (ΔCt-values > 0: lower abundance with phenotype, ΔCt-values < 0: higher abundance with phenotype). (A) *hsa*-miR-885-5p, (B) *hsa*-miR-199a-5p, (C) *hsa*-miR-194-5p.

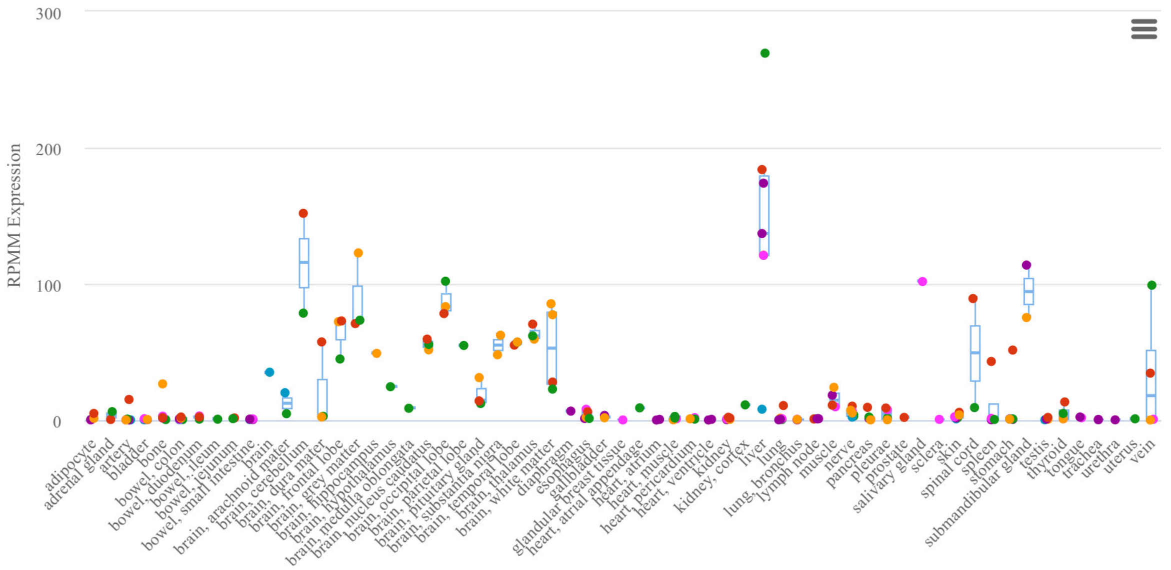

RPMM Expression
300
200
100
0
adipocyte
adrenal gland
artery
bladder
bone
bowel, colon
bowel, duodenum
bowel, ileum
bowel, jejunum
bowel, small intestine
brain
brain, arachnoid mater
brain, cerebellum
brain, dura mater
brain, frontal lobe
brain, grey matter
brain, hippocampus
brain, hypothalamus
brain, medulla oblongata
brain, nucleus caudatus
brain, occipital lobe
brain, parietal lobe
brain, pituitary gland
brain, substantia nigra
brain, temporal lobe
brain, thalamus
brain, white matter
diaphragm
esophagus
gallbladder
glandular breast tissue
heart, atrial appendage
heart, atrium
heart, muscle
heart, pericardium
heart, ventricle
kidney
kidney, cortex
liver
lung
lung, bronchus
lymph node
muscle
nerve
pancreas
pleurae
prostate
salivary gland
sclera
skin
spinal cord
spleen
stomach
submandibular gland
testis
thyroid
tongue
trachea
urethra
uterus
vein

A

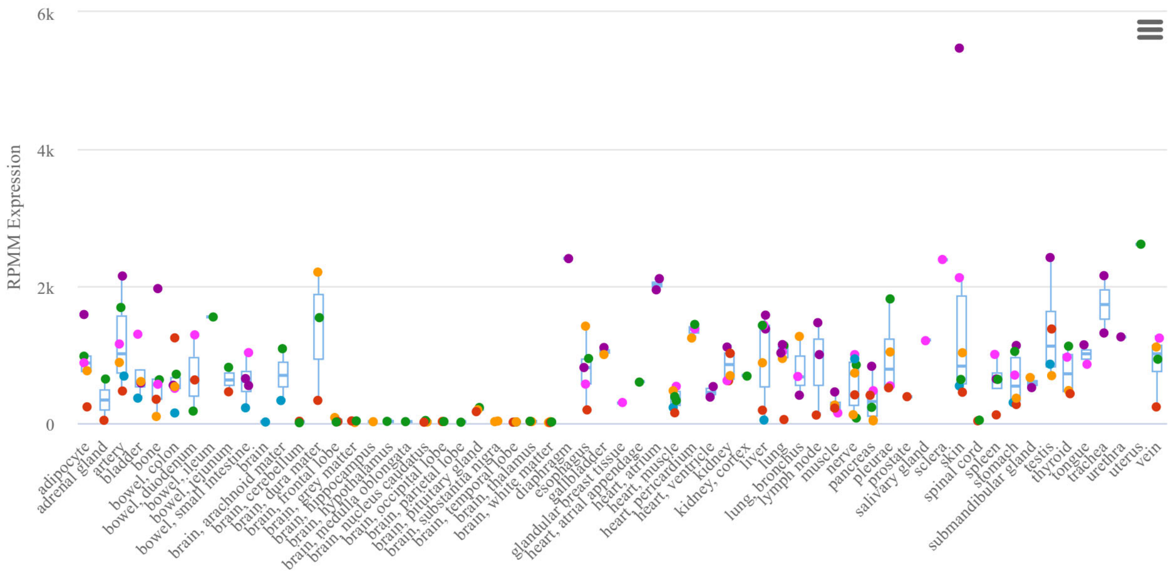

RPMM Expression
6k
4k
2k
0
adipocyte
adrenal gland
artery
bladder
bone
bowel, colon
bowel, duodenum
bowel, ileum
bowel, jejunum
bowel, small intestine
brain
brain, arachnoid mater
brain, cerebellum
brain, dura mater
brain, frontal lobe
brain, grey matter
brain, hippocampus
brain, hypothalamus
brain, medulla oblongata
brain, nucleus caudatus
brain, occipital lobe
brain, parietal lobe
brain, pituitary gland
brain, substantia nigra
brain, temporal lobe
brain, thalamus
brain, white matter
diaphragm
esophagus
gallbladder
glandular breast tissue
heart, atrial appendage
heart, atrium
heart, muscle
heart, pericardium
heart, ventricle
kidney
kidney, cortex
liver
lung
lung, bronchus
lymph node
muscle
nerve
pancreas
pleurae
prostate
salivary gland
sclera
skin
spinal cord
spleen
stomach
submandibular gland
testis
thyroid
tongue
trachea
urethra
uterus
vein

B

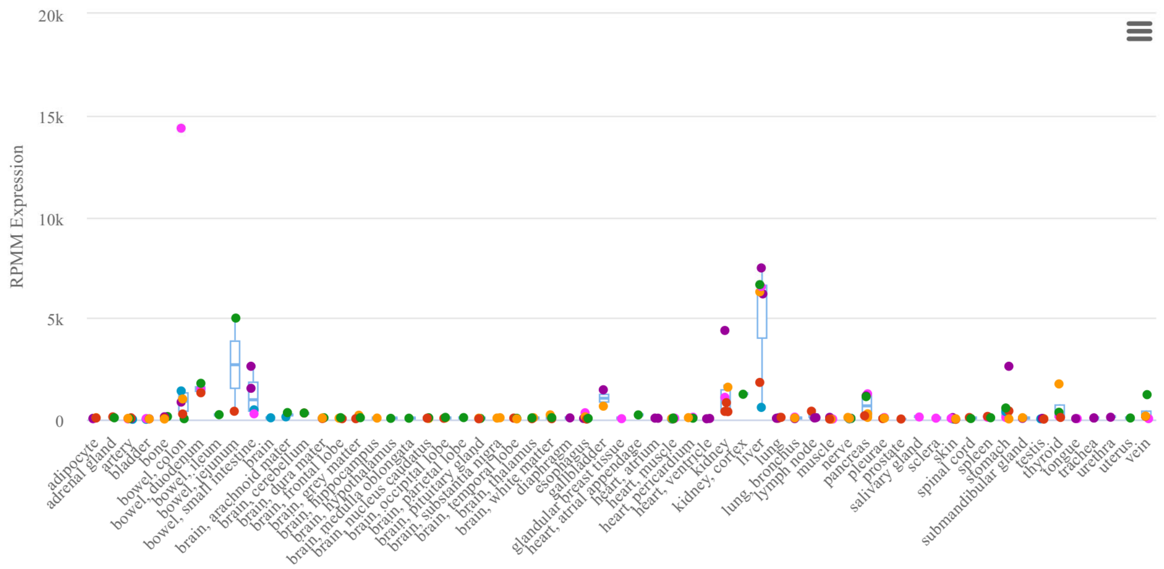

C

Figure S20. A. Expression of miRNA *hsa*-miR-885-5p significant in the smoking interaction model in different tissues (https://ccb-web.cs.uni-saarland.de/tissueatlas2/). **B.** Expression of miRNA *hsa*-miR-199a-5p significant in the smoking interaction model in different tissues (https://ccb-web.cs.uni-saarland.de/tissueatlas2/). **C.** Expression of miRNA *hsa*-miR-194-5p significant in the smoking interaction model in different tissues (https://ccb-web.cs.uni-saarland.de/tissueatlas2/).
